# Supplementary material for: An exact chiral amorphous spin liquid
Source: Nat Commun. 2023 Oct 20;14:6663. doi: 10.1038/s41467-023-42105-9 (PMC10589230; doi:10.1038/s41467-023-42105-9)
Supplement: Supplementary file 1 — Supplementary Information [file 41467_2023_42105_MOESM1_ESM.pdf]

Supplementary Material: An Exact Chiral Amorphous Spin Liquid

G. Cassella [1,*] P. D'Ornellas [1,*] T. Hodson [1,*] W. M. H. Natori [2] and J. Knolle [1,3,4]

[1] *Blackett Laboratory, Imperial College London, London SW7 2AZ, United Kingdom*
[2] *Institut Laue-Langevin, BP 156, 41 Avenue des Martyrs, 38042 Grenoble Cedex 9, France*
[3] *Department of Physics TQM, Technische Universität München, James-Franck-Straße 1, D-85748 Garching, Germany*
[4] *Munich Center for Quantum Science and Technology (MCQST), 80799 Munich, Germany*

I. LATTICE GENERATION

We generate tri-coordinate lattices by taking the Voronoi partition of the unit square with respect to uniformly sampled *seed points* [1]. This partitions the space into polyhedral volumes enclosing the region closest to each seed point. In two dimensions, the vertices and edges of these polygons form a tri-coordinate lattice, exactly what is necessary for the Kitaev model. To produce lattices with periodic boundary conditions we first tile the seed points into a repeating lattice. The Voronoi partition of the tiled seed points can then be converted into a lattice embedded onto the torus by connecting corresponding edges that cross the unit square boundaries, see fig. 1. Finally, to somewhat regularise bond lengths in our lattice, a single step of Lloyd's algorithm is performed, where every vertex is shifted to the center of mass of the three plaquettes that it touches. This is done to improve the readability of the lattice, reducing the number of extremely short bonds appearing, and has no effect on the physics.

Once a lattice has been generated, the bonds must be labelled in such a way that no vertex touches multiple edges of the same type, which we refer to as a *three-edge colouring*. The problem of finding such a colouring is equivalent to the classical problem of four-colouring the faces, which is always solvable on a planar graph [2, 3]. On the torus, a face colouring can require up to seven colours [4], so not all graphs can be assumed to be 3-edge colourable. However, such exceptions seem rare for graphs generated via voronisation – every graph generated in this study admitted multiple distinct 3-edge colourings. In practice, the problem of finding a colouring for a given graph can be reduced to a Boolean satisfiability problem [5], which we then solve using the open-source solver `MiniSAT` [6].

Care must be taken in the definition of open boundary conditions, simply removing bonds from the lattice leaves behind unpaired b^α operators that need to be paired in some way to arrive at fermionic modes. In order to fix a pairing we always start from a lattice defined on the torus and generate a lattice with open boundary conditions by defining the bond coupling $J^\alpha_{ij} = 0$ for sites joined by bonds (i, j) that we want to remove. This creates fermionic zero modes u_{ij} associated with these cut bonds which we set to 1 when calculating the projector. All our code is available online [7].

II. THE PROJECTOR

Closely following the derivation of [8] we can extend the projector from Majorana wavefunctions to physical spin states to the amorphous case. In the standard way, we define normal mode operators

$$(c_1, c_2 ... c_{2N})Q = (b_1, b'_1, b_2, b'_2 ... b_N, b'_N)$$

such that the Hamiltonian comes into the form

$$\tilde{H}_u = \frac{i}{2} \sum_m \epsilon_m b_m b'_m$$

from there we form fermionic operators $f_i = \frac{1}{2}(b_m + ib'_m)$ and their associated number operators $n_i = f_i^\dagger f_i$. The many body ground state within a vortex sector is then defined by the set of occupation numbers $n_m = 0, 1$. Lastly we need to define the fermion parity $\hat{\pi} = \prod i^N (1 - 2\hat{n}_i)$.

The projector can be written as

$$\mathcal{P} = \mathcal{S} \left(\frac{1 + \prod_i^{2N} D_i}{2} \right) = \mathcal{S} \cdot \mathcal{P}_0$$

where D_i are the local projectors. $\mathcal{S}$ symmetrises over gauge equivalent states while $\mathcal{P}_0$ is responsible for annihilating unphysical states, see [8] for details.

To extend this to the amorphous case we calculate the product of the local projectors D_i

$$\prod_i^{2N} D_i = \prod_i^{2N} b_i^x b_i^y b_i^z c_i$$

for a tri-coordinate lattice with N faces, $2N$ vertices and $3N$ edges. The operators can be ordered by bond type without utilising any property of the lattice.

$$\prod_i^{2N} D_i = \prod_i^{2N} b_i^x \prod_i^{2N} b_i^y \prod_i^{2N} b_i^z \prod_i^{2N} c_i$$

The product over c_i operators reduces to a determinant of the Q matrix and the fermion parity. The only problem

* These three authors contributed equally, and names are ordered alphabetically.

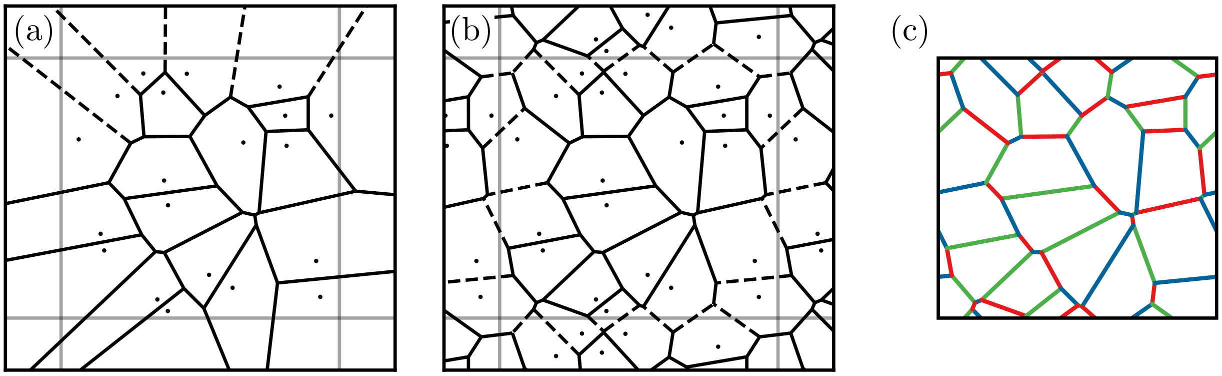

Supplementary Figure 1. The Voronoi Construction: **(a)** The Voronoi partition (lines) splits a region up into polyhedra closer to one of the seed points (points) than any other. In two dimensions this yields a tri-coordinate lattice. Dotted lines go off to infinity. **(b)** To create a lattice on the torus, we tile the seed points into a 3x3 grid and compute a Voronoi partition. By identifying pairs of edges (dotted lines) that cross the unit square (in grey) as the same we turn this lattice into on defined on the torus. **(c)** The final tri-coordinate lattice in periodic boundary conditions, coloured such that all three colours meet at every vertex.

is to compute the factors $p_x, p_y, p_z = \pm 1$ that arise from reordering the b operators such that pairs of vertices linked by the corresponding bonds are adjacent.

$$\prod_i^{2N} b_i^\alpha = p_\alpha \prod_{(i,j)} b_i^\alpha b_j^\alpha$$

This is simple the parity of the permutation from one ordering to the other and can be computed quickly with a cycle decomposition.

The final form is almost identical to the honeycomb case with the addition of the lattice structure factors p_x, p_y, p_z

$$P^0 = 1 + p_x\ p_y\ p_z \det(Q^u)\ \hat{\pi} \prod_{\{i,j\}} -iu_{ij},$$

where $\det(Q^u)$ and $\prod u_{ij}$ depend on the lattice and the particular vortex sector.

III. NUMERICAL EVIDENCE FOR THE GROUND STATE FLUX SECTOR

In this section we detail the numerical evidence collected to support the claim that, for an arbitrary lattice, a gapped ground state flux sector is found by setting the flux through each plaquette to $\phi_{\text{g.s.}} = -(\pm i)^{n_{\text{sides}}}$. This was done by generating a large number ($\sim$ 25,000) of lattices and exhaustively checking every possible flux sector to find the configuration with the lowest energy. We checked both the isotropic point ($J^\alpha = 1$), as well as in the toric code ($J^x = J^y = 0.25, J^z = 1$).

The argument has one complication: for a graph with n_p plaquettes, there are $2^{n_p - 1}$ distinct flux sectors to search over, with an added factor of 4 when the global fluxes Φ_x and Φ_y wrapping around the cylinder directions are taken into account. Note that the -1 appears in this counting because fluxes can only be flipped in pairs. To be able to search over the entire flux space, one is necessarily restricted to looking at small system sizes – we were able to check all flux sectors for systems with $n_p \leq 16$ in a reasonable amount of time. However, at such small system size we find that finite size effects are substantial. In order to overcome these effects we tile the system and use Bloch's theorem (a trick that we shall refer to as *twist-averaging* for reasons that shall become clear) to efficiently find the energy of a much larger (but periodic) lattice. Thus we are able to suppress finite size effects, at the expense of losing long-range disorder in the lattice.

Our argument has three parts: First we shall detail the techniques used to exhaustively search the flux space for a given lattice. Next, we discuss finite-size effects and explain the way that our methods are modified by the twist-averaging procedure. Finally, we demonstrate that as the size of the disordered system is increased, the effect of twist-averaging becomes negligible – suggesting that our conclusions still apply in the case of large disordered lattices.

Testing All Flux Sectors — For a given lattice and flux sector, defined by $\{u_{jk}\}$, the fermionic ground state energy is calculated by taking the sum of the negative eigenvalues of the matrix

$$M_{jk} = \frac{i}{2} J^\alpha u_{jk}. \tag{1}$$

The set of bond variables u_{jk}, which we are free to choose, determine the $\mathbb{Z}_2$ gauge field. However only the fluxes,

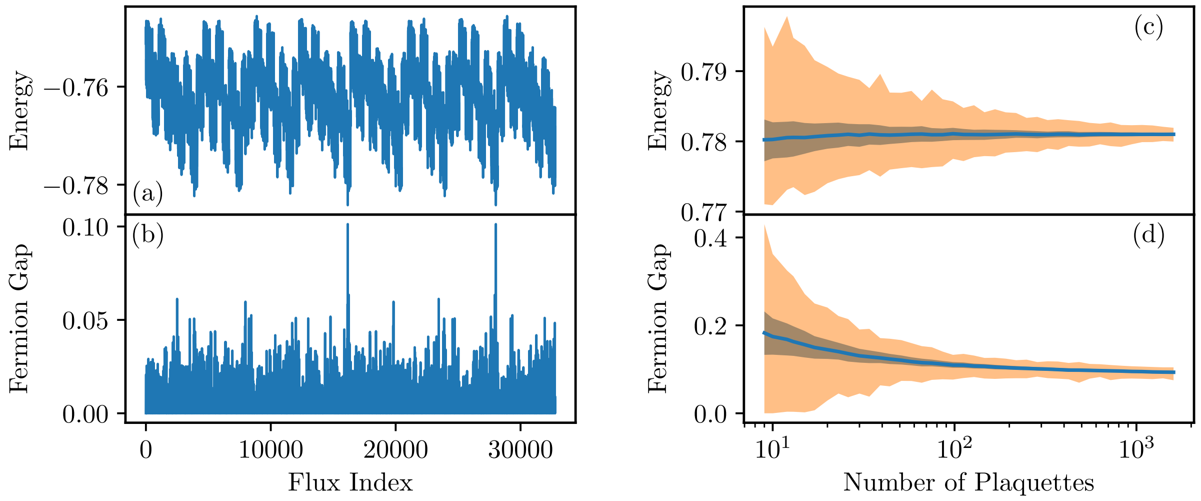

Supplementary Figure 2. Evidence for the Ground State Flux Sector: **(a)** The energy of every flux sector explored for a system of 16 plaquettes, the order is arbitrary. The two ground state flux sectors can be identified as the points with lowest energy. **(b)** The fermion gap for each of the flux sectors explored. Note that the largest fermion gap coincides with the ground state flux sector. This occurred in $\sim 85\%$ of cases tested. (c) Average energy of the systems tested over a range of system sizes from $n_p = 9$ to $n_p = 1600$. The region between the upper and lower quartiles is shown in red, and the full range of energies obtained is shown in orange. (d) Average fermion gap as a function of system size. Again, the region between the upper and lower quartiles is shown in red, and the full range is shown in orange. As can be seen, no gapless systems were found for $n_p > 20$.

defined for each plaquette according to

$$\phi_p = \prod_{(j,k)\in\partial p} -iu_{jk}, \tag{2}$$

have any effect on the energies. Thus, there is enormous degeneracy in the u_{jk} degrees of freedom. Flipping the bonds along any closed loop on the dual lattice has no effect on the fluxes, since each plaquette has had an even number of its constituent bonds flipped - as is shown in the following diagram:

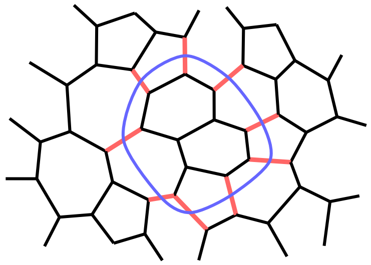

where the flipped bonds are shown in red. In order to explore every possible flux sector using the u_{jk} variables, we restrict ourselves to change only a subset of the bonds in the system. In particular, we construct a spanning tree on the dual lattice, which passes through every plaquette in the system, but contains no loops.

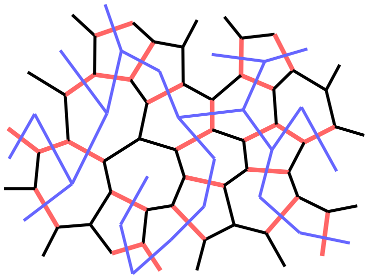

The tree contains $n_p - 1$ edges, shown in red, whose configuration space has a $1:1$ mapping onto the 2^{n_p-1} distinct flux sectors. Each flux sector can be created in precisely one way by flipping edges only on the tree (provided all other bond variables not on the tree remain fixed). Thus, all possible flux sectors can be accessed by iterating over all configurations of edges on this spanning tree.

Finite Size Effects — In our numerical investigation, the objective was to test as many example lattices as possible. We aim for the largest lattice size that could be efficiently solved, requiring a balance between lattice size and cases tested. Each added plaquette doubles the number of flux sectors that must be checked. 25,000 lattices containing 16 plaquettes were used. However, in his numerical investigation of the honeycomb model, Kitaev demonstrated that finite size effects persist up to much larger lattice sizes than we were able to access [9].

In order to circumvent this problem, we treat the 16-plaquette amorphous lattice as a unit cell in an arbitrarily large periodic system. The bonds that originally connected across the periodic boundaries now connect ad-

jacent unit cells. This infinite periodic Hamiltonian can then be solved using Bloch's theorem, since the larger system is diagonalised by a plane wave ansatz. For a given crystal momentum $\mathbf{q} \in [0, 2\pi)^2$, we are left with a Bloch Hamiltonian, which is identical to the original Hamiltonian aside from an extra phase on edges that cross the periodic boundaries in the x and y directions,

$$M_{jk}(\mathbf{q}) = \frac{i}{2} J^{\alpha} u_{jk} e^{i q_{jk}}, \tag{3}$$

where $q_{jk} = q_x$ for a bond that crosses the x-periodic boundary in the positive direction, with the analogous definition for y-crossing bonds. We also have $q_{jk} = -q_{kj}$. Finally $q_{jk} = 0$ if the edge does not cross any boundaries at all – in essence we are imposing twisted boundary conditions on our system. The total energy of the tiled system can be calculated by summing the energy of $M(\mathbf{q})$ for every value of $\mathbf{q}$. In practice we constructed a lattice of 50×50 values of $\mathbf{q}$ spanning the Brillouin zone. The procedure is called twist averaging because the energy-per-unit cell is equivalent to the average energy over the full range of twisted boundary conditions.

Evidence for the Ground State Ansatz — For each lattice with 16 plaquettes, $2^{15} = 32{,}768$ flux sectors are generated. In each case we find the energy (averaged over all twist values) and the size of the fermion gap, which is defined as the lowest energy excitation for any value of $\mathbf{q}$. We then check if the lowest energy flux sector aligns with our ansatz (given by $\phi_p^{\text{g.s.}} = -(\pm i)^{n_{\text{sides}}}$) and whether this flux sector is gapped.

In the isotropic case ($J^{\alpha} = 1$), all 25,000 examples conformed to our guess for the ground state flux sector. A tiny minority (~ 10) of the systems were found to be gapless. As we shall see shortly, the proportion of gapless systems vanishes as we increase the size of the amorphous lattice. An example of the energies and gaps for one of the systems tested is shown in fig. 2 (a). For the anisotropic phase (we used $J^x, J^y = 0.25, J^z = 1$) the overwhelming majority of cases adhered to our ansatz, however a small minority ($\sim 0.5\%$) did not. In these cases, however, the energy difference between our ansatz and the ground state was at most of order 10^{-6}. Further investigation would need to be undertaken to determine whether these anomalous systems are a finite size effect due to the small amorphous system sizes used or a genuine feature of the toric code phase on such lattices.

A Gapped Ground State — Now that we have collected sufficient evidence to support our guess for the ground state flux sector, we turn our attention to checking that this sector is gapped. We no longer need to exhaustively search over flux space for the ground state, so it is possible to go to much larger system size. We generate 40 sets of systems with plaquette numbers ranging from 9 to 1600. For each system size, 1000 distinct lattices are generated and the energy and gap size are calculated without phase twisting, since the effect is negligible for such large system sizes. As can be seen in fig. 2 (c-d), for very small system size a small minority of gapless systems appear, however beyond around 20 plaquettes all systems had a stable fermion gap in the ground state. Finally, the energy and gap difference between the phase twisted and non-phase twisted results vanished exponentially with the system size, supporting the claim that the results can be straightforwardly extrapolated to large systems.

IV. THE EFFECT OF BOND DISORDER

The effect of bond disorder in the Kitaev honeycomb lattice model is well studied [10–12]. When bond disorder is introduced the fermionic gap closes as a function of disorder strength, leading generically to a thermal metal phase [10, 12]. The changes to the fermionic spectrum of the flux-free sector are much more dramatic than those observed in our amorphous model. *A priori* one might expect that a similar gap closing should be introduced by the lattice disorder present in the amorphous model. As we have shown, this is not the case. We believe the essential difference between these kinds of disorder is the disruption of the local motif: in the amorphous model, the global translational symmetry of the system is disrupted, but the coupling strengths remain locally homogeneous on each site.

We study the effect of bond disorder on the amorphous lattice by introducing a bond-length dependent factor to the hopping terms in the Hamiltonian, such that $J_{jk} = J^0_{jk}/r^{\alpha}_{jk}$, where r_{jk} is the bond length from site j to site k, which has been normalised such that $\langle r^{\alpha}_{jk} \rangle = 1$ to prevent this term from substantially rescaling the energies of the system. The parameter α controls the strength of the disorder, where setting $\alpha = 0$ reproduces the original Hamiltonian. As α is varied, we calculate the fermionic spectrum, observing how the band gap is affected by this disorder.

Clearly, this Hamiltonian will produce unphysical results on any lattice where some bonds are extremely short-ranged, leading to extremely strong hopping terms. Thus, in order to get physical results, one must first regularise the bond lengths of the lattice. This is done in two steps. First, the initial points are sampled from a blue noise distribution, rather than a uniform distribution, using Mitchell's best-candidate algorithm [13, 14]. This produces seed points that are regularly spaced, yet still amorphous. The second step is to regularise the lattice using Lloyd's algorithm [15] – where vertices are repeatedly moved to the centre of mass of the plaquettes adjacent and the Voronoi diagram is regenerated. In practice we used four iterations of this method. An example of such a lattice is shown in fig. 3 (b).

The fermionic spectrum is calculated for a range of α between 0 and 8, at a variety of lattice sizes parametrised by the length L, where the number of vertices N scales with $N \sim L^2$. As disorder is increased, the gap shrinks however no truly gapless systems are generated for systems sizes larger than $L \sim 10$, providing evidence of the Kitaev phase's stability in the presence of even relatively strong

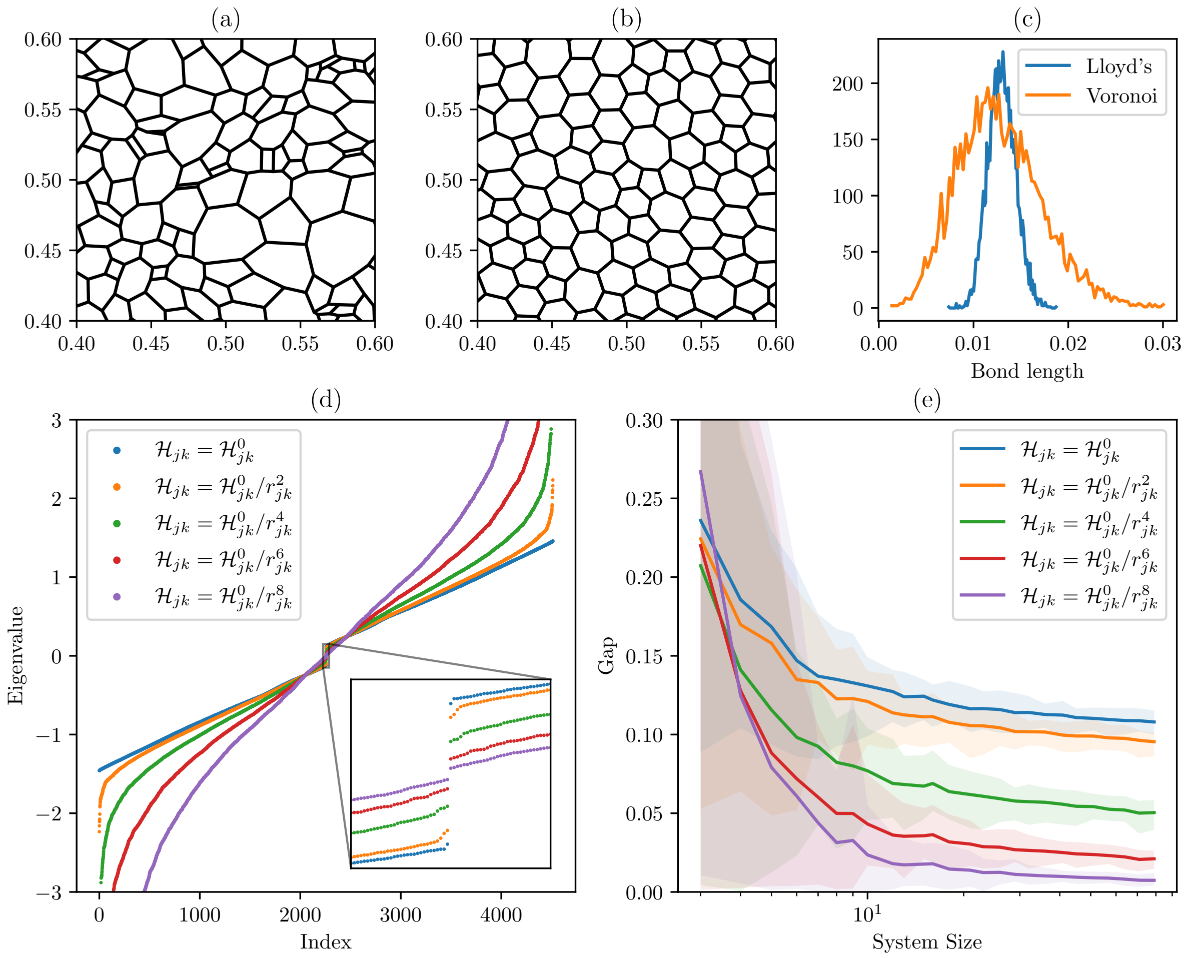

Supplementary Figure 3. The Effect of Bond Disorder: **(a)** Close up of a section of amorphous lattice generated using a single voronoi partition on a set of uniformly sampled seed points. **(b)** A section of amorphous lattice generated using the regularised method – we create a Voronoi partition using seed points sampled from a blue noise distribution. This is then followed by four iterations of Lloyd's algorithm to further regularise the bond lengths. **(c)** A histogram of bond lengths for the example graphs in sub figures a and b. Note that the distribution is much more tightly peaked around the mean bond length in the regularised lattice, with no extremely short bonds present. **(d)** The Majorana spectrum for a single example of an amorphous lattice for five different values of $\alpha \in [0, 8]$. **(e)** Finite-size scaling analysis of the fermion gap in the flux-free sector. Solid lines indicate the gap averaged over 50 random lattice samples for each system size, and the shaded regions indicate the full range of the sampled gaps.

bond disorder.We note that these calculations were carried out in periodic boundary conditions, hence the absence of in-gap edge states.

V. EVIDENCE FOR AN ANDERSON TRANSITION TO A THERMAL METAL PHASE

Here we present numerical evidence to support the claim that, as temperature is increased, the gapped chiral spin liquid undergoes an Anderson transition to a gapless thermal metal phase [12, 16, 17]. As discussed in the bulk text, we look for two signatures of this transition: a closing of the fermion gap driven by the flux sectors with a gapless fermionic spectrum, and the characteristic low energy oscillations in the density of states predicted by random matrix theory (RMT) [12, 18].

We study the closing of the fermion gap using the flux density ρ as a proxy for temperature. This approximation is exact in the limits $T = 0$ ($\rho = 0$) and $T \to \infty$ ($\rho = 0.5$). At intermediate temperatures the method neglects the influence of flux-flux correlations. However, we are only interested in whether the gap closes at all. The fermionic density of states as a function of ρ is shown in fig. 4(a). As the defect density increases, the gap becomes populated with fermionic states. We quantify the degree to which a state is localised by calculating the dimensional scaling exponent of the IPR with the linear extent of the system, $L \sim \sqrt{N}$, with N being the number of sites on the lattice,

$$\mathrm{IPR} \propto L^{-\tau}. \tag{4}$$

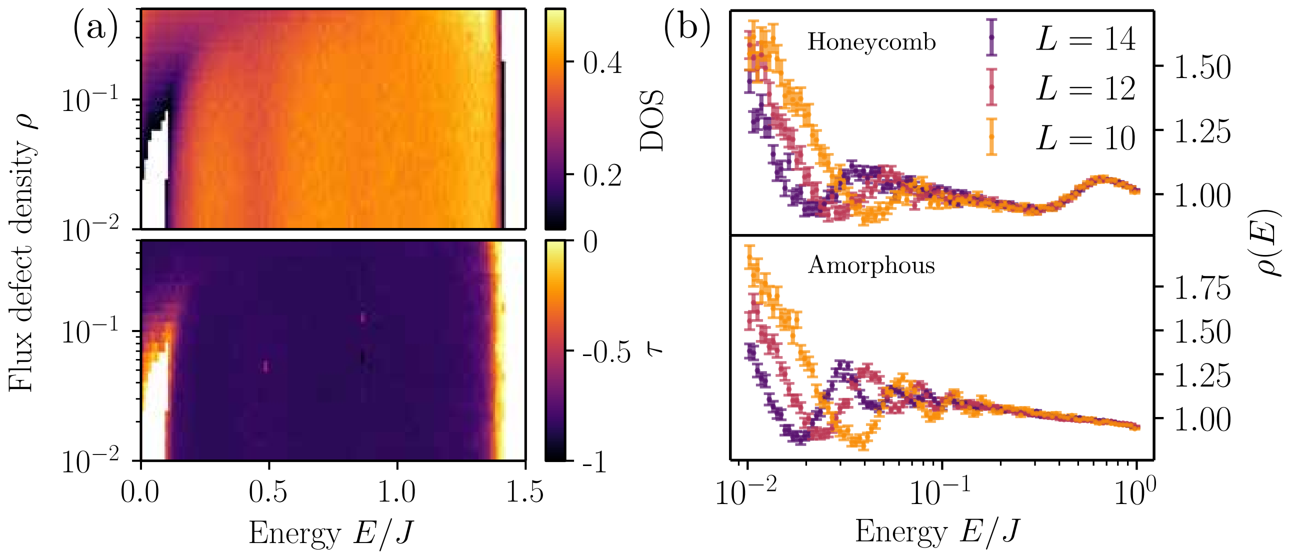

Supplementary Figure 4. Anderson Transition to a Thermal Metal: **(a)** Density of states (top) and inverse participation ratio scaling exponent (bottom) of the fermionic spectrum as a function of flux defect density, ρ, for isotropic couplings. Each pixel is averaged over 10 independent lattice realisations, in flux sectors sampled from an ensemble with a proportion ρ of fluxes flipped with respect to the ground state sector. White pixels correspond to bins containing no fermionic states. At low defect density, the fermionic spectra are gapped. As the defect density increases, in-gap states appear with a small τ, indicating that they are strongly localized around defects. At large defect density, τ increases for the in-gap sites, indicating that they are delocalised and the system becomes gapless. Using defect density as a proxy for temperature, this demonstrates the thermal phase transition from a chiral spin liquid to a thermal metal phase. **(b)** A histogram of fermionic density of states sampled from the thermodynamic ensemble of flux sectors for $T \to \infty$, i.e. all gauge configurations equally likely. The oscillations at low E are characteristic of a thermal metal phase [12], demonstrated for the Kitaev honeycomb lattice model subject to a magnetic field (top) and the amorphous Kitaev model (bottom). L corresponds to the linear extent of the system – $L \sim \sqrt{N}$ with N sites – for both lattice types.

At small ρ, the states populating the gap possess $\tau \approx 0$, indicating that they are localised states pinned to individual fluxes – the system remains insulating. At larger ρ, the in-gap states merge with the bulk band and become extensive, closing the gap – the system transitions to a metallic phase.

Finally, the averaged density of states in the $\rho = 0.5$ case is shown in fig. 4 (b) for both the Honeycomb model and our amorphous lattice. Note that only the honeycomb model is calculated in the presence of an effective magnetic field explicitly breaking TRS. In both cases we see the logarithmic scaling alongside the characteristic RMT oscillations at low energy, giving strong evidence that the amorphous model displays a finite temperature transition to a thermal metal phase.

[1] M. Florescu, S. Torquato, and P. J. Steinhardt, Proceedings of the National Academy of Sciences **106**, 20658 (2009).

[2] P. G. Tait, Proceedings of the Royal Society of Edinburgh **10**, 501 (1880).

[3] K. Appel and W. Haken, *Every Planar Map Is Four Colorable*, Contemporary Mathematics, Vol. 98 (American Mathematical Society, 1989).

[4] G. Ringel and J. W. Youngs, Proceedings of the National Academy of Sciences of the United States of America **60**, 438 (1968).

[5] R. M. Karp, Reducibility among combinatorial problems, in *Complexity of Computer Computations*, edited by R. E. Miller, J. W. Thatcher, and J. D. Bohlinger (Springer US, Boston, MA, 1972) pp. 85–103.

[6] A. Ignatiev, A. Morgado, and J. Marques-Silva, in *SAT* (2018) pp. 428–437.

[7] T. Hodson, P. d'Ornellas, and G. Cassella, Imperial-CMTH/koala (2022).

[8] F. L. Pedrocchi, S. Chesi, and D. Loss, Phys. Rev. B **84**, 165414 (2011).

[9] A. Kitaev, Annals of Physics January Special Issue, **321**, 2 (2006).

[10] J. Knolle, *Dynamics of a Quantum Spin Liquid* (Springer, 2016).

[11] J. Knolle, R. Moessner, and N. B. Perkins, Phys. Rev. Lett. **122**, 047202 (2019).

[12] C. N. Self, J. Knolle, S. Iblisdir, and J. K. Pachos, Phys. Rev. B **99**, 045142 (2019).

[13] D. P. Mitchell, in *Proceedings of the 18th Annual Confer-*

ence on Computer Graphics and Interactive Techniques, SIGGRAPH '91 (Association for Computing Machinery, New York, NY, USA, 1991) p. 157–164.

[14] demofox2, Generating Blue Noise Sample Points With Mitchell's Best Candidate Algorithm (2017).

[15] S. Lloyd, IEEE Transactions on Information Theory **28**, 129 (1982).

[16] C. R. Laumann, A. W. W. Ludwig, D. A. Huse, and S. Trebst, Phys. Rev. B **85**, 161301(R) (2012).

[17] J. T. Chalker, N. Read, V. Kagalovsky, B. Horovitz, Y. Avishai, and A. W. W. Ludwig, Phys. Rev. B **65**, 012506 (2001).

[18] M. Bocquet, D. Serban, and M. R. Zirnbauer, Nuclear Physics B **578**, 628 (2000).
